# Primary Blast-Induced Changes in Akt and GSK_3β_ Phosphorylation in Rat Hippocampus

**DOI:** 10.3389/fneur.2017.00413

**Published:** 2017-08-18

**Authors:** Yushan Wang, Thomas W. Sawyer, Yiu Chung Tse, Changyang Fan, Grant Hennes, Julia Barnes, Tyson Josey, Tracy Weiss, Peggy Nelson, Tak Pan Wong

**Affiliations:** ^1^Defence Research and Development Canada, Suffield Research Centre, Medicine Hat, AB, Canada; ^2^Department of Psychiatry, Douglas Mental Health University Institute, McGill University, Montreal, QC, Canada

**Keywords:** traumatic brain injury, Akt, primary blast, *N*-methyl-d-aspartate receptor, neurotrauma

## Abstract

Traumatic brain injury (TBI) due to blast from improvised explosive devices has been a leading cause of morbidity and mortality in recent conflicts in Iraq and Afghanistan. However, the mechanisms of primary blast-induced TBI are not well understood. The Akt signal transduction pathway has been implicated in various brain pathologies including TBI. In the present study, the effects of simulated primary blast waves on the phosphorylation status of Akt and its downstream effector kinase, glycogen synthase kinase 3β (GSK_3β_), in rat hippocampus, were investigated. Male Sprague-Dawley (SD) rats (350–400 g) were exposed to a single pulse shock wave (25 psi; ~7 ms duration) and sacrificed 1 day, 1 week, or 6 weeks after exposure. Total and phosphorylated Akt, as well as phosphorylation of its downstream effector kinase GSK_3β_ (at serine 9), were detected with western blot analysis and immunohistochemistry. Results showed that Akt phosphorylation at both serine 473 and threonine 308 was increased 1 day after blast on the ipsilateral side of the hippocampus, and this elevation persisted until at least 6 weeks postexposure. Similarly, phosphorylation of GSK_3β_ at serine 9, which inhibits GSK_3β_ activity, was also increased starting at 1 day and persisted until at least 6 weeks after primary blast on the ipsilateral side. In contrast, p-Akt was increased at 1 and 6 weeks on the contralateral side, while p-GSK_3β_ was increased 1 day and 1 week after primary blast exposure. No significant changes in total protein levels of Akt and GSK were observed on either side of the hippocampus at any time points. Immunohistochemical results showed that increased p-Akt was mainly of neuronal origin in the CA1 region of the hippocampus and once phosphorylated, the majority was translocated to the dendritic and plasma membranes. Finally, electrophysiological data showed that evoked synaptic *N*-methyl-d-aspartate (NMDA) receptor activity was significantly increased 6 weeks after primary blast, suggesting that increased Akt phosphorylation may enhance synaptic NMDA receptor activation, or that enhanced synaptic NMDA receptor activation may increase Akt phosphorylation.

## Introduction

The increased use of improvised explosive devices (IEDs) in recent wars and in acts of terrorism is a major cause of casualties (1–4). Nevertheless, due to advances in personal protection equipment, the survival rate after major blast events has increased dramatically in the past decade. However, those who survive often suffer from traumatic brain injury (TBI) (5). These injuries can be immediately obvious, or occur in a delayed fashion, where individuals who initially exhibited no immediate trauma show signs of cognitive impairments and behavioral changes weeks or even months after being exposed to blast (1, 6, 7). For this reason, mild traumatic brain injury (mTBI) caused by IEDs has been recognized as one of the most significant injuries of recent conflicts in Afghanistan and Iraq.

The exact mechanisms by which primary blast waves cause brain damage are still under investigation (8–16). Recent reports suggest that primary waves can directly affect brain tissues (17, 18); while others believe that blast-induced TBI is mainly caused by acceleration/deceleration, at least in animal models (19). It has also been suggested that a blast wave can be transmitted to the brain through a thoracic mechanism (18, 20). However, these possibilities do not mutually exclude each other. In addition to brain injury caused by the blast wave, victims can also be struck by penetrating fragments, or thrown by the blast wind, resulting in secondary or tertiary injuries, respectively (21). Regardless of how blast waves cause brain damage, animal models that reliably reproduce clinical features of TBI are important in the understanding of its pathology and in helping to develop standard treatments for TBI (20).

In primary blast-induced TBI (PbTBI), the hippocampus, the frontal cortex, and the cerebellum exhibit variable degrees of damage after blast exposure (22, 23). Diffuse axonal injury, subdural hemorrhage, hypoxia, and ischemia have all been observed in moderate to severe cases (22, 24). This damage leads to biochemical and metabolic changes that eventually result in tissue damage, changes in neuronal adhesion, and synaptic transmission (25), and associated neuronal degeneration, but not necessarily cell death (26). In addition to neurodegeneration, gliosis in the forms of microglia activation and/or astrocyte proliferation in the brain has been demonstrated to exist in various models of TBI (14), most likely as a result of inflammatory responses to damage (14, 20, 27). Astrocyte proliferation as measured by the enhanced expression of glial fibrillary acidic protein (GFAP) has also been suggested as occurring after PbTBI, although this is not a consistent finding (17, 28).

Akt, also known as protein kinase B, is a serine/threonine protein kinase that is ubiquitously expressed in all tissues. In the central nervous system, Akt plays important roles in neuronal development, cell proliferation, damage repair, and cell survival (29). Under normal circumstances, Akt is expressed at low levels in the brain, both in unphosphorylated and phosphorylated forms. However, its phosphorylation levels are altered in both neurons and glial cells during cellular stress, brain inflammation, and various forms of brain injury (30–33). In order for Akt to exert its functions, it must be activated through phosphorylation at both threonine-308 and serine-473 positions *via* its upstream kinase phosphatidylinositol 3-kinase (PI3K) in response to various growth and survival factors such as insulin-like growth factor (34) or to the activation of synaptic *N*-methyl-d-aspartate (NMDA) receptors (35, 36). Upon phosphorylation, Akt is translocated to the cell membrane to modulate its downstream effectors including glycogen synthase kinase-3β (GSK_3β_), which plays an important role in regulating neurodegeneration during disease and injury (37). Previous reports have shown that Akt and its downstream effector kinase GSK_3β_ activities are differentially regulated by TBI in animal models, with Akt phosphorylation being depressed, while GSK_3β_ activity is upregulated (31, 38). In contrast, a previous study using controlled cortical impact (CCI) as a TBI model in rats showed that Akt phosphorylation was increased 3 days post-injury (39). While in another study, GSK_3β_ phosphorylation at serine 9 was transiently increased at 3 days post-injury, and GSK_3β_ inhibitors successfully reversed pathological changes after CCI in rats (40). Importantly, recent studies with various posttraumatic stress disorder (PTSD) models have also shown that the phosphorylation of Akt at both serine 473 and threonine 308 are consistently increased after chronic stress, and that this increase contributes to the behavioral changes observed in these animal models (4, 41). However, the role of Akt and GSK_3β_ phosphorylation in blast-induced TBI has not been previously reported.

This laboratory has established an animal model of PbTBI (17). The present study has utilized this model system to investigate the molecular mechanisms that may be involved in PbTBI. We hypothesize that the phosphorylation of Akt, as well as its downstream effector kinase, GSK_3β_, are differentially regulated in rat hippocampus after primary blast exposure. The hippocampus was chosen as the region of interest in this study because it is considered one of the most vulnerable regions in the brain in response to damage such as brain ischemia. This is mainly due to its unique anatomical characteristics where the majority of neurons in this region are densely packed in a single layer and more prone to oxidative stress than neurons in other areas (42). Furthermore, the hippocampus performs important cognitive functions such as learning and memory (43), and memory deficits are common in TBI and PTSD patients (44).

## Materials and Methods

### Primary Blast Exposure

All experimental procedures in this study were carried out according to the “Guide to the Care and Use of Experimental Animals” and “The Ethics of Animal Experimentation” published by the Canadian Council on Animal Care, and were approved by the Animal Care Committee at DRDC Suffield Research Centre. A total of 48 adult male Sprague-Dawley rats were acquired from Charles River Laboratories (St. Constant, QC, Canada) and acclimated for at least 1 week prior to exposure. Exposure of animals to primary blast has been described previously (17). Briefly, on the day of use, the animals (~280–330 g) were anesthetized with 3% isoflurane in oxygen and placed into a restraint consisting of a clear plastic cylindrical sleeve, with the neck encircled snugly in a plastic collar and the head protruding from the end. The hind quarters were supported using an end cap fitted with a piston. On the right side of the head, mesh netting was secured between two pins placed vertically in line with the side of, and above and below the head. The head was placed against this vertical netting, and then restrained using additional netting around the head. After 8 min of anesthesia, the restraint containing the animal was set into the wall of the Advanced Blast Simulator (ABS), 4.28 m downstream from the diaphragm, such that only the head protruded into the test section (17). Test groups consisted of sham control, and head-only, side-on exposures of single pulse shock waves of 25 psi static overpressure. Animals were sacrificed at 1 day, 1 week, or 6 weeks after blast exposure and their brains harvested. Animals were divided into control and blast groups with each group containing 5 rats for each time point studied, with the exception of 1 week after blast groups, where 10 rats for each group were used (5 in each group for immunohistochemistry). At the time of harvest, the animals were anaesthetized with isoflurane in an anaesthetization chamber. For experiments involving western blot analysis, animals (five control and five blasted) were sacrificed by decapitation after anaesthetization and their brains quickly removed and placed in ice cold dissecting buffer [CelLytic™, Sigma-Aldrich Canada, Oakville, ON, Canada to which protease inhibitors (Roche Complete) had been added, pH 7.4]. Both the ipsilateral and contralateral sides (relative to shock wave propagation) of the hippocampi were separated under a dissecting microscope, and lysates were prepared by homogenizing the brain tissue with a Teflon-glass tissue homogenizer (Eberbach Corp., Ann Arbor, MI, USA) in approximately 10 volumes of CelLytic™ cell lysis buffer and stored at −80°C until analysis. For experiments involving immunohistochemistry, 1 week after blast exposure, animals (five in each group) were anaesthetized with isoflurane in an anaesthetization chamber followed by intraperitoneal injection of 1 ml Euthanyl (240 mg/ml) and immediately perfused transcardially with 100 ml of 0.1 M phosphate-buffer saline (PBS, pH 7.4) followed by 4% paraformaldehyde (approximately 500 ml). The brains were removed and post-fixed with 4% paraformaldehyde for 48 h at room temperature. The fixed brains were then immersed in 30% sucrose in PBS in a 50 ml falcon tube for dehydration and cytoprotection at 4°C until they sank to the bottom of the tube. Once at the bottom of the tube, the brains were blotted dry with a tissue paper and frozen in dry ice, then stored at −80°C until use.

### Western Blot Analysis

Brain homogenate protein lysates (10–15 µg protein) from either side of the hippocampi were separated on a 4–20% gradient precast gel (Bio-Rad Laboratories, Mississauga, ON, Canada) and transferred onto polyvinylidene difluoride membranes. The membranes were blocked with 2% ECL blocking powder (Bio-Rad Laboratories), 0.1% Tween 20 in PBS, and then incubated with antibodies against phospho-Akt Ser^473^, total Akt, phospho-GSK_3β_ Ser^9^, or total GSK_3β_ (1:1,000, New England Biolabs, Whitby, ON, Canada) overnight at 4°C. The membranes were then washed with PBST (PBS plus 0.1% Tween 20) three times at 10 min each. Primary antibodies were probed by incubating membranes with a secondary antibody; donkey anti-rabbit IgG-HRP (GE Health Care Biosciences, QC, Canada) diluted 1:5,000. Detection was carried out by using ECL advanced detection reagents (GE Health Care Biosciences) and imaged using a Molecular Imager Versa Doc MP 4000 system (Bio-Rad Laboratories). After probing with phospho-Akt or phospho-GSK_3β_ antibodies, the membranes were stripped using a stripping buffer (Restore™ Western Blot Stripping Buffer, Rockford, IL, USA) for 10 min and re-probed with total Akt or GSK_3β_ antibodies (1:1,000). To confirm equal protein loading, blots were stripped again and re-probed with anti-tubulin antibody (1:50,000; New England Biolabs). Band intensities were quantified using Image Lab software (Bio-Rad Laboratories) and expressed as percentage of control. At least six samples were analyzed from each group and results were expressed as mean ± SEM.

### Immunohistochemistry and Confocal Microscopy

Coronal sections of fixed brains containing the hippocampus (30 µm thickness) were prepared with a Cryostat (Leica Microsystems Canada, Concord, ON, Canada). Frozen sections of hippocampal slices were rehydrated in PBS for at least 30 min at room temperature before being stained. After rehydration, antigen retrieval was achieved by treating the slices in acetic acid buffer (pH 6.0) at 80°C in a pressure cooker (Decloaking chamber, Biocare Medical, Concord, CA, USA) for 35 min. The slices were then permeabilized with 0.20% triton X-100 for 1 h and blocked in 5% goat serum for 1 h before being stained with the primary antibodies. Hippocampal slices were immunostained with phospho-Akt Ser^473^ (1:50, New England Biolabs), NeuN (1:200, Millipore Ltd., Toronto, ON, Canada), or microtubule-associated protein 2 (MAP-2, 1:100, Sigma-Aldrich). Stained primary antibodies were detected with Alexa Fluor-488 or Alexa Fluor-647 tagged secondary antibodies (Thermo Fisher Scientific, Burlington, ON, Canada). At the end of the staining process, all slices were counter-stained with DAPI (Thermo Fisher Scientific) to view nuclei. Stained brain slices were viewed with a Quorum WaveFX laser scanning confocal microscope (Quorum Technologies Inc., Guelph, ON, Canada) at 400× magnification and images were captured with a Hamamatsu EM-CCD camera. Finally, captured images were stitched together using a module from MetaMorph to show structures of different brain regions. Areas of interest were then cropped to include approximately 500 µm sections for presentation.

### Hippocampal Slice Preparation and Field Recording

Animals were exposed to primary blast (25 psi, *n* = 4) at DRDC Suffield Research Centre and observed for 4 weeks prior to shipping to the Douglas Mental Health University Institute, Montreal, QC, Canada where electrophysiology was performed. Both control and blast-exposed animals were housed in a quarantine facility and observed for two more weeks before being used. At the day of electrophysiological recording, brains were rapidly removed from decapitated rats 6 weeks after primary blast exposure, and coronal brain slices (400 µm thickness) were cut in hyperosmotic, ice-cold and carbogenated slice cutting solution (in mM: 252 sucrose, 2.5 KCl, 4 MgCl_2_, 0.1 CaCl_2_, 1.25 KH_2_PO_4_, 26 NaHCO_3_, and 10 glucose, ~360 mOsmol/l) using a vibratome. Freshly cut slices were incubated with carbogenated artificial cerebrospinal fluid (aCSF in mM: 125 NaCl, 2.5 KCl, 1 MgCl_2_, 2 CaCl_2_, 1.25 NaH_2_PO_4_, 26 NaHCO_3_, and 25 glucose, ~310 mOsmol/l) at 32°C for 1 h and subsequently maintained at room temperature. Bicuculline methobromide (10 µM, Sigma) was used to block GABA_A_ receptor-mediated inhibitory synaptic transmission in all recordings. Postsynaptic responses were evoked by stimulating the Schaffer collateral-commissural pathway [constant current pulses (0.08 ms) through a tungsten bipolar electrode (FHC)] and recorded in the hippocampal CA1 stratum radiatum. Synaptic responses were amplified and digitized by Multiclamp 700B and Digidata 1,400, respectively (Molecular Devices, Sunnyvale, CA, USA), and stored in a PC for offline analysis using Clampfit (Molecular Devices). All recordings were performed at room temperature.

Postsynaptic responses were evoked at 0.05 Hz. Field excitatory postsynaptic potentials (fEPSPs) were detected using CSF-filled glass electrodes. To isolate NMDAR-mediated fEPSPs (NMDAR-fEPSP), field recording was performed in the presence of low Mg^2+^ (0.05 mM) aCSF in the presence of an AMPAR antagonist DNQX (20 µM, Sigma).

### Statistical Analysis

Statistical differences were analyzed using two-way ANOVA and Sidak’s multiple comparisons test.

## Results

### Simulated Blast Exposure

Animals were exposed to single pulse shock waves at target static overpressures of 25 psi. The actual pressures measured were 25.7 ± 0.5 psi with a positive duration of 7.46 ± 0.34 ms and a positive phase impulse of 54.6 ± 1.4 psi.ms. Dynamic pressure values were not assessed during these experiments, but have been previously recorded in the ABS as 13.8 ± 0.6 psi for 25 psi overpressures (17).

### Primary Blast Increases Phosphorylation of Akt and GSK_3β_

**Figure 1:**
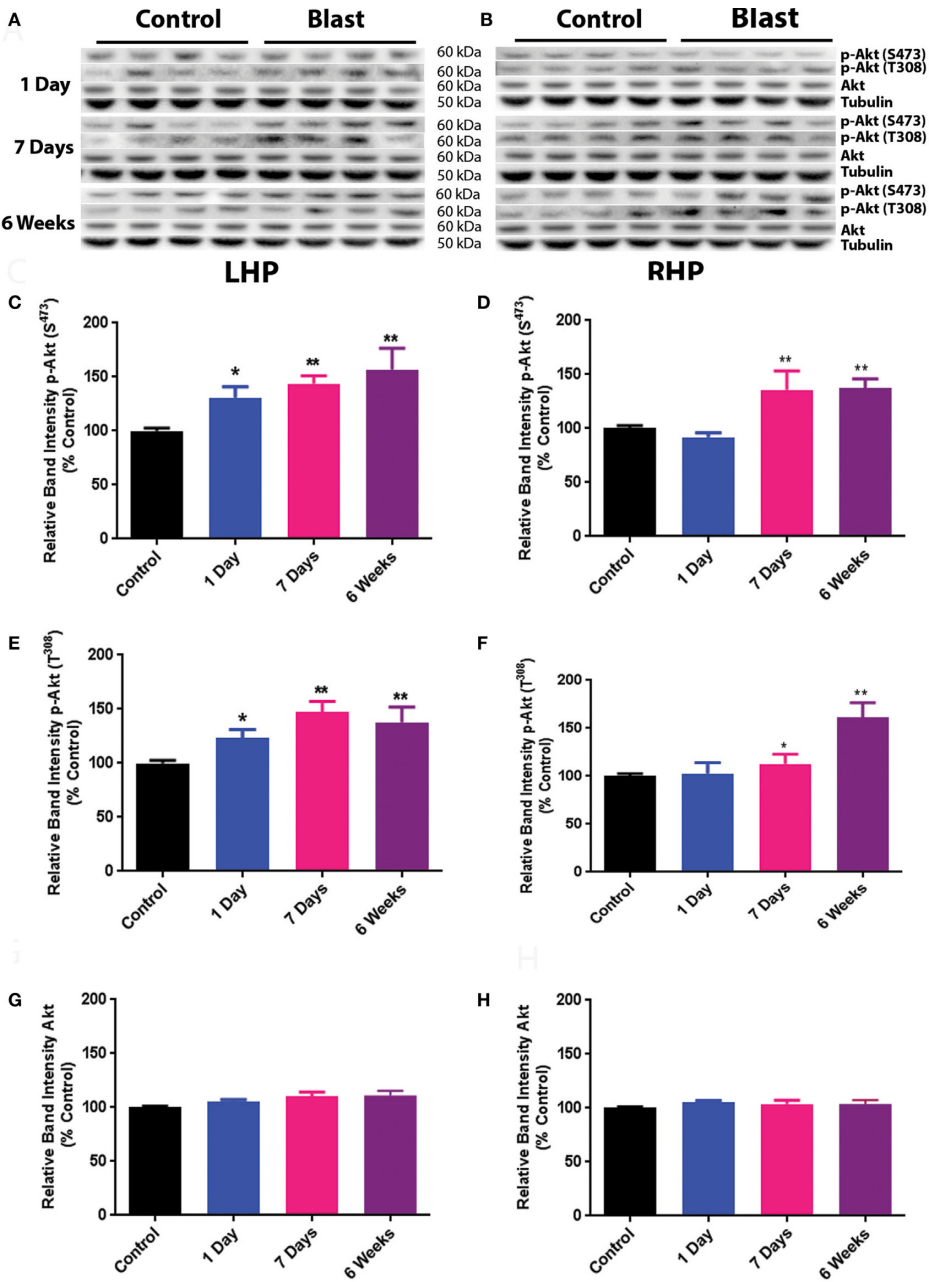
Effect of primary blast (25 psi) on Akt phosphorylation in the rat hippocampus. Data show the results obtained from the ipsilateral **(A,C,E,G)** and contralateral **(B,D,F,H)** sides of the hippocampus relative to shock wave exposure. **(A,B)** Representative western blot bands from control and blasted rat hippocampal homogenates. Note that p-Akt S^373^ and p-Akt T^308^ western blot bands were not from the same membrane. Total Akt and tubulin bands were from one of the membranes that were probed with either p-Akt S^473^ or p-Akt T^308^ antibodies. Each lane under both control and blast represents a separate animal in their respective groups. **(C,D)** Relative band intensity of p-Akt S^473^ at various times after primary blast exposure. **(E,F)** Relative band intensity of p-Akt T^308^ at various times after primary blast exposure. **(G,H)** Relative band intensity of total Akt at various times after primary blast exposure. Mean ± SEM from at least four different rats. *P < 0.05, **P < 0.01, compared with control. LHP, left hippocampus; RHP, right hippocampus.

The effects of primary blast-induced changes on the phosphorylation of Akt in rat hippocampus are shown in Figure 1. No significant changes in the expression of total Akt were observed on both ipsilateral (left hippocampus, LHP) and contralateral (right hippocampus, RHP) sides of the hippocampus at any time point after primary blast exposure (Figures 1A,B,G,H). However, the phosphorylation of Akt at both serine 473 and threonine 308 was significantly elevated on the ipsilateral side of the hippocampus 1 day after primary blast as compared to control (*P* < 0.05) and remained elevated at 1 and 6 weeks after blast (Figures 1C,E). Compared to the ipsilateral side, Akt phosphorylation at both sites on the contralateral side was not changed at 1 day but increased at 1 and 6 weeks after primary blast exposure (Figures 1D,F).

**Figure 2:**
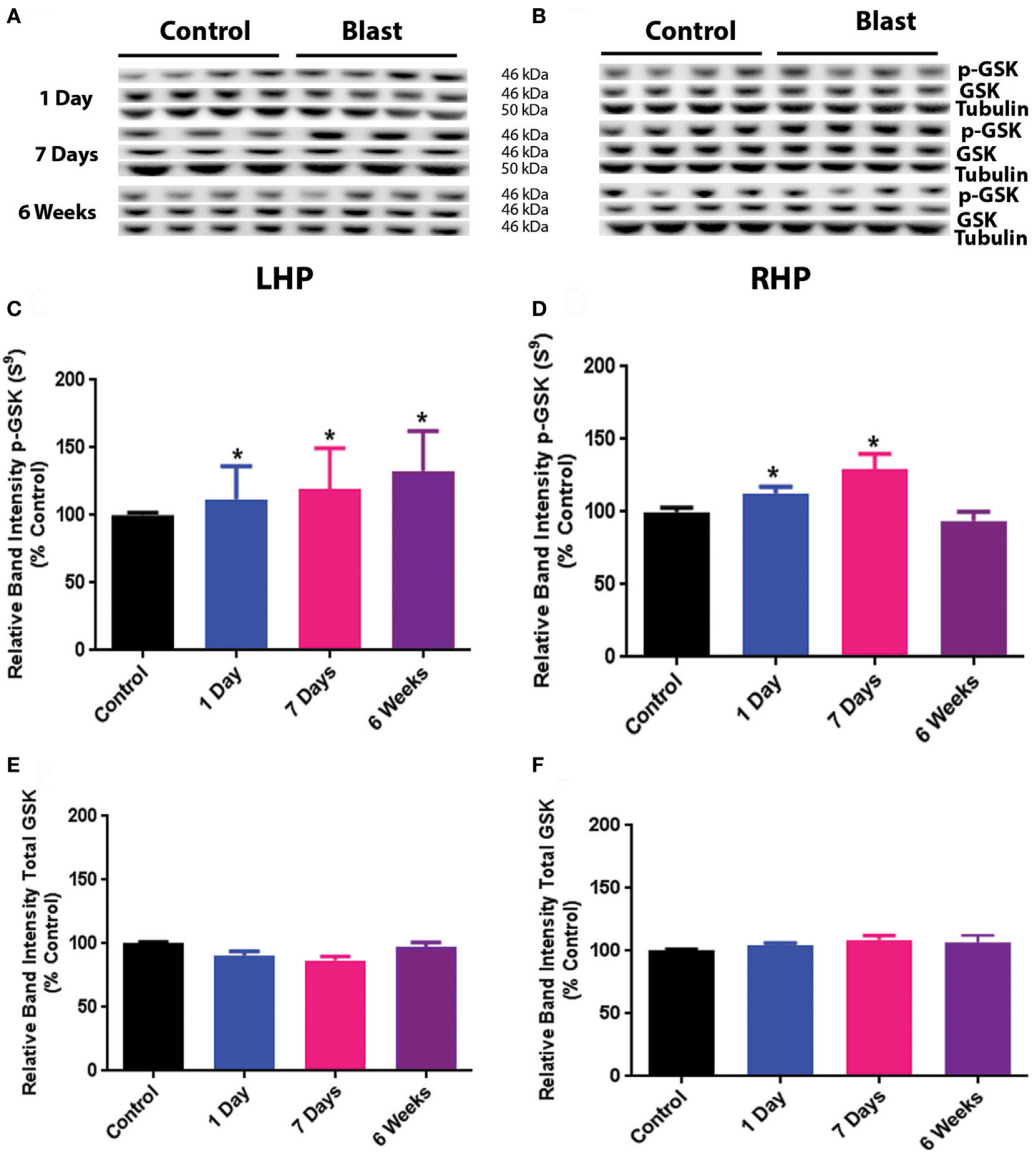
Effect of primary blast (25 psi) on GSK_3β_ phosphorylation in the rat hippocampus. Data show the results obtained from the ipsilateral **(A,C,E)** and contralateral **(B,D,F)** sides of the hippocampus relative to shock wave exposure. **(A,B)** Representative western blot bands from control and blasted rat hippocampal homogenates. Each lane under both control and blast represents a separate animal in their respective groups. **(C,D)** Relative band intensity of pGSK_3β_ S^9^ at various times after primary blast exposure. **(C,F)** Relative band intensity of total GSK_3β_ at various times after primary blast exposure. Mean ± SEM from at least four different rats. *P < 0.05, **P < 0.01, compared with control. LHP, left hippocampus; RHP, right hippocampus.

The results for GSK_3β_ phosphorylation at Serine 9 after primary blast are shown in Figure 2. Similar to the effect of primary blast on Akt phosphorylation, there was no significant difference in total GSK_3β_ protein expression on either side of the hippocampus at any time point after primary blast exposure (Figures 2A,B,E,F). However, phosphorylation of GSK_3β_ at Serine 9 was significantly increased at 1 day, 1 week, and 6 weeks on the ipsilateral side after primary blast exposure of the hippocampus (Figures 2C,D), indicating a decrease in GSK_3β_ activity. Increased phosphorylation of GSK_3β_ Serine 9 was also observed on the contralateral side of the hippocampus at 1 day and 1 week after primary blast exposure.

### Phosphorylated Akt Is Located in Hippocampal CA1 Neurons and Translocated to Cell Membranes

**Figure 3:**
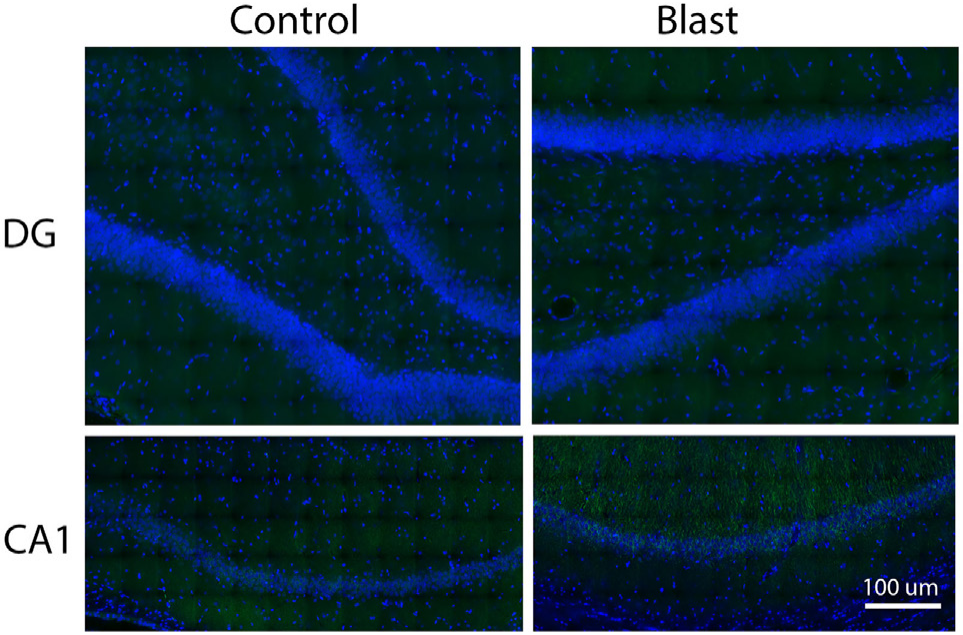
Immunohistochemical staining of p-Akt S^473^ in the dentate gyrus (DG) and CA1 regions of issilateral hippocampus 1 week after primary blast (25 psi) exposure. Phospho Akt was shown in green. No apparent positive staining was observed in the DG area. DAPI was used to counterstain nuclei (blue). Magnification: 400×.

**Figure 4:**
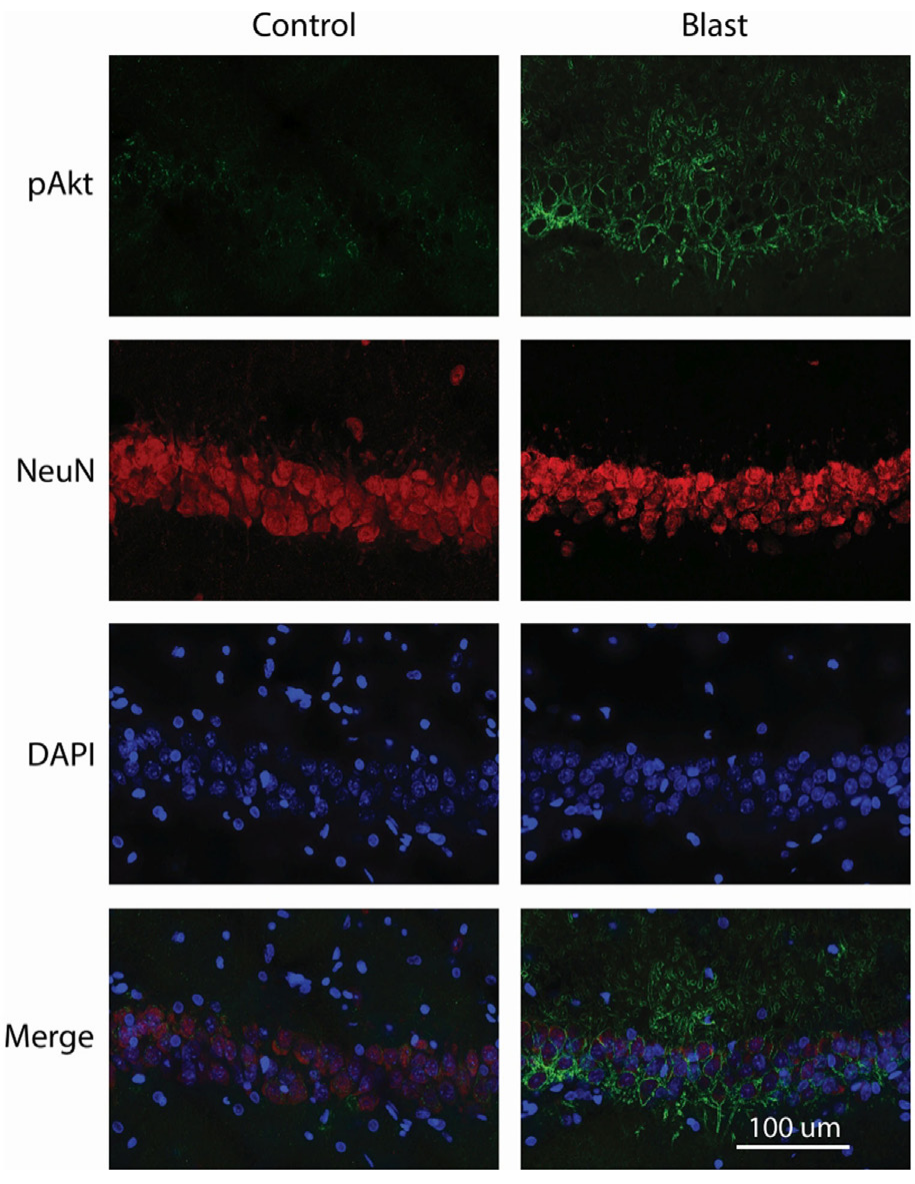
Co-immunostaining of p-Akt S^473^ and NeuN in the CA1 region of ipsilateral hippocampus 1 week after primary blast (25 psi) exposure. Left panels depict representative images from sham control animals, while the right panels show images from primary blast-exposed animals. Phosphorylated Akt is shown in green and NeuN in red. Phosphorylated Akt colocalizes with NeuN. DAPI was used to counterstain nuclei (blue). Magnification: 400×.

We next examined the localization of phosphorylated Akt in the ipsilateral side of the hippocampus using fluorescence immunohistochemistry and confocal microscopy. After staining with anti-phospho-Akt (S473) antibody, both the dentate gyrus (DG) and the CA1 regions of the hippocampus were scanned. However, only the CA1 region showed positive staining in both control and primary blast exposed brain slices (Figure 3). Therefore, only the CA1 region was presented for subsequent studies to investigate the localization of phospho Akt. Figure 4 shows that the fluorescence intensity of phospho-Akt is increased compared to control at 1 week after primary blast exposure in the CA1 region of the ipsilateral hippocampus. Moreover, the increased phospho-Akt was mainly expressed in CA1 neurons, as stained with the neuronal nuclei marker NeuN, indicating that phosphorylated Akt is primarily of neuronal origin in the CA1 region of the hippocampus.

**Figure 5:**
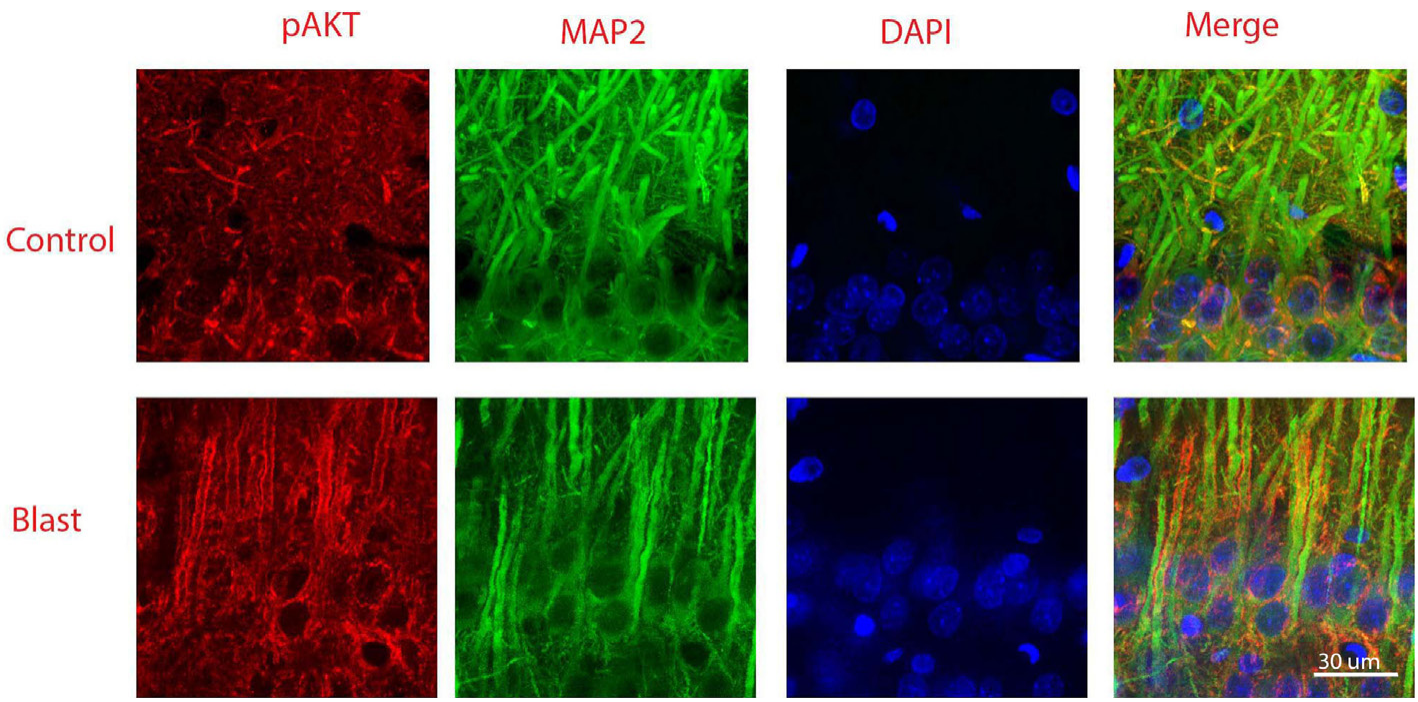
Co-immunostaining of pAkt S^473^ and microtubule-associated protein 2 (MAP-2) in the CA1 region of ipsilateral hippocampus 1 week after primary blast (25 psi) exposure. Phosphorylated Akt was shown in red and MAP-2 was shown in green. Phosphorylated Akt is mainly stained along dendritic membranes. DAPI was used to counterstain nuclei (blue). Magnification: 400×.

Figure 5 shows double staining of phospho-Akt and the neuronal dendritic marker MAP-2 at 1 week after primary blast exposure in the CA1 region of the ipsilateral hippocampus. As shown in the merged image, low levels of phospho-Akt were mainly distributed in the cytosol in the control group. In contrast, the majority of the increased phospho-Akt was redistributed to plasma and dendritic membranes after primary blast, suggesting translocation. However, the distribution of the increased phospho-Akt was not exclusively dendritic, as can been seen in the merged figure of the blast group (Figure 5), where some of the phospho-Akt staining did not completely colocalize with MAP-2.

### Primary Blast Activates Synaptic NMDA Receptors

**Figure 6:**
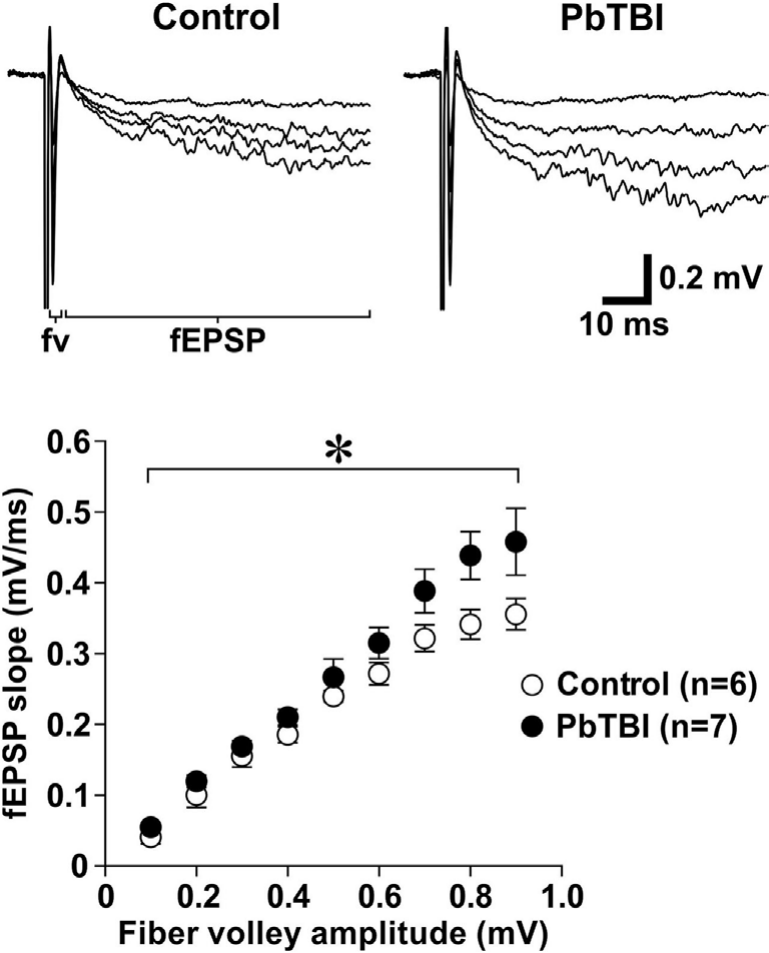
Effect of primary blast (25 psi) on synaptic *N*-methyl-d-aspartate (NMDA) receptor function in the hippocampal CA1 region. Representative traces of NMDA receptor-mediated fEPSP recorded from control (*n* = 6; 3 rats) and primary blast-induced TBI rats (6 weeks after blast injury, *n* = 7; 4 rats). Scatter plots below show the relationship between fiber volley amplitude and the slope of NMDA receptor-mediated fEPSP. *P < 0.05, group effect from repeated measures (fiber volley amplitudes) ANOVA.

To investigate the effect of primary blast on glutamate neurotransmission in the hippocampus, fEPSPs were recorded from live brain slices from control rats and rats at 6 weeks after primary blast exposure (PbTBI). Two-way repeated measures ANOVA (fiber volley amplitudes as repeated measures) showed that fEPSP slope was significantly increased after primary blast [*F*(8, 88) = 2.28; *P* = 0.029, Figure 6], indicating increased synaptic NMDA receptor activity.

## Discussion

In this study, an ABS was used to simulate free-field blast waves with high fidelity, including sharply defined static and dynamic overpressure rise times, underpressures, and secondary shock waves (17). The pressure used (25 psi) in this study was based on previous work (17), where rats were head-only exposed to overpressures of 15–30 psi and duration times of ~6.1–7.5 ms. These exposures are approximately equivalent to a 25 kg surface burst at 8.0 m, which the Conventional Weapons Effects (Conwep) software code (45) predicts will yield a peak incident pressure of 168.5 kPa (24.44 psi), with a duration of 7.38 ms. According to Bowen et al. (46), this blast would cause less than 1% mortality and be just above threshold lung injury. However, while these conditions would not cause significant movement of the human head, this was not the case with the relatively small rat head. As a consequence, when the head was unrestrained, exposures to overpressures (15–30 psi) at these durations (6.1–7.5 ms) resulted in the head experiencing exaggerated global motion, resulting in significant elevation of endpoints such as GFAP. In contrast, when head movement was minimized, GFAP elevation was dramatically reduced (17). These studies point to the difficulties of scaling small rodent studies to humans. At this juncture and in this study, we significantly reduce the exaggerated head motion artifact by utilizing head restraint during the head-only simulated blast exposures. However, ongoing work is developing ABS capabilities with which to expose small rodents to similar overpressures, but scaled to humans by reducing the peak pressure durations.

Head-only exposure of rats to nominal 25 psi overpressure caused significant increases in the phosphorylation of Akt and its downstream effector kinase GSK_3β_ in both the ipsilateral and contralateral sides of the rat hippocampus. These changes were observed as early as 1 day and persisted up to 6 weeks post-exposure. The choice of time points in this study is arbitrary. Ideally, a time course of injury in an animal model should relate to the development of neuropathological symptoms in human subjects. However, no clear link has been established in the progression of injury between small animals and clinical cases. Future studies will investigate the pathological consequences of PbTBI immediately after exposure (e.g., 30 min) as well as long-term effects up to 1 year post-exposure.

The PI3K–Akt–GSK_3_ signal transduction pathway has been implicated in a variety of brain disorders, including various forms of brain injuries such as brain ischemia and TBI (31, 47, 48). The activation of Akt plays a pivotal role in enhancing learning and memory, promoting cell survival, and inhibiting apoptosis after damage. In order for Akt to be active, it requires phosphorylation at both serine 473 and threonine 308. Upon phosphorylation, Akt is translocated to the cell membranes, where it inhibits the activity of GSK_3β_ *via* phosphorylation of the latter at serine 9 (47). In a previous report, Noshita et al. demonstrated that the phosphorylation of Akt is rapidly (1 h after impact) decreased in the injured cortex while being elevated in the peripheral tissues (4 h after impact), before returning to control levels by 24 h in a mouse model of cortical impact TBI (31). In the present study, we found that the phosphorylation of Akt was increased in rat hippocampus at 1 day after primary blast, and this increase persisted for at least 6 weeks post exposure. The increase in Akt phosphorylation is somewhat surprising because previous reports have shown declines in Akt phosphorylation after other types of brain injury (31, 49). However, the nature of primary blast is very different than the impact/acceleration types of insult that other animal models of TBI (e.g., CCI, fluid percussion) use. Notably, primary blast would not be expected to include the focal damage that is seen in these other model systems. In addition, our exposure regimen simulates only low-level blast exposure and only mild TBI effects would be expected to result in our model system (17). This is further demonstrated by recent findings in our laboratories (50), as well as in others (51, 52), that primary blast does not cause cell death in *in vitro* models of primary blast.

The serine/threonine protein kinase GSK 3 also plays important roles in neurodegeneration caused by various pathological conditions such as TBI and neurodegenerative diseases (53, 54). One of the most important upstream regulators of GSK 3 activity is the PI3K–Akt pathway. Upon phosphorylation, Akt inhibits GSK 3 activity by phosphorylating the latter at serine 9. It is interesting to note that, while at most time points, the increase in Akt phosphorylation is coupled with the increase in GSK_3β_ phosphorylation in LHP, this relationship is less clear in RHP. This is particularly noticeable at 6 weeks after primary blast exposure in RHP, where Akt phosphorylation was dramatically increased whereas that of GSK_3β_ showed a slight decrease, though that decrease was not statistically significant. This suggests that Akt perhaps is not the exclusive regulator of GSK_3β_ activity after primary blast, and that GSK_3β_ activity may increase long term after exposure. Thus, we speculate that GSK_3β_ inhibitors may be suitable for the treatment of PbTBI in the longer term, similar to TBI induced by other models (54). However, this hypothesis needs to be investigated further in future studies.

In addition to brain injury and neurodegenerative diseases, Akt has also been reported to contribute to symptoms of PTSD. It is estimated that approximately 15% of individuals who are exposed to traumatic events or severe stress will ultimately develop PTSD (55). Symptoms include chronic re-experiencing of the traumatic events (e.g., nightmares of battlefield situations in returned veterans from the war zone), hyperarousal (e.g., startle response to a normal noise), and avoidance behaviors. The exact mechanisms of PTSD remain to be elucidated. Previously, in a rat model of PTSD, Eagle et al. (56) reported that a single prolonged stress enhances Akt phosphorylation in the hippocampus, which contributes to the increased response to fear conditioning. Fear conditioning-related memory is the hallmark of PTSD in animal models. Therefore, the delayed enhancement in Akt phosphorylation observed in our study may contribute to PTSD-like symptoms after PbTBI. This may also provide a mechanistic link between PbTBI and PTSD. However, due to the lack of behavioral data in this study, the role of increased Akt phosphorylation in the development of PTSD-like symptoms remains speculative. Nevertheless, the increase in Akt phosphorylation can still serve as a biomarker for primary blast-induced mTBI.

The underlying mechanism(s) upon which Akt is activated is complex. One of the most important upstream events that activate PI3K and its subsequent phosphorylation of Akt is the activation of synaptic NMDA receptors (57). It has long been established that calcium influx upon synaptic NMDA receptor activation induces the phosphorylation of cyclic-AMP response element-binding protein, which in turn activates PI3K and its downstream effector kinases, including Akt. Once phosphorylated, Akt further phosphorylates its downstream effector kinase, GSK_3β_, at serine 9, which inhibits GSK_3β_ activity (47). The ultimate consequence of this cascade of events is enhancement of learning and memory (58), which may contribute to the development of PTSD-like symptoms in PbTBI. Furthermore, since the Akt pathway is involved in preventing cell death and promoting cell survival, the increase in Akt phosphorylation observed in this study may contribute to the activation of the intrinsic neuroprotective mechanism against further damage after primary blast. The observation that evoked synaptic NMDA receptor responses were increased in a long-term fashion in the hippocampus after primary blast exposure indicate that the increased phosphorylation of Akt observed in this study may be a result of synaptic NMDA receptor activation. Alternatively, the observed increase in Akt phosphorylation may have an impact on NMDA receptors, which leads to a long lasting increase in NMDA receptor function. For instance, the increase in NMDA receptor function caused by brain-derived neurotrophic factor, which is known to trigger activation of various kinase pathways including the Akt pathway, can be abolished by Akt inhibitors (59). In addition, Akt has been shown to phosphorylate GluN1 (60) and GluN2B subunits (61) of NMDA receptors on spinal cord neurons. Considering the importance of glutamate neurotransmission in the brain and the prevalence of TBI and PTSD, the relationship between Akt and synaptic NMDA receptor activation after PbTBI warrants further investigation.

In conclusion, significant increases in Akt phosphorylation were observed in rat hippocampus after primary blast-induced mild TBI in our study. Whereas changes in Akt phosphorylation may not be specific to PbTBI, it may nevertheless contribute to PTSD-like symptoms after primary blast. Therapeutics targeting this signal transduction pathway may help alleviate anxiety and fear-related symptoms caused by PTSD or primary blast. However, a definitive role of enhanced Akt phosphorylation in the development of PTSD-like symptoms requires further experiments involving behavioral analysis and employment of Akt inhibitors.

## Ethics Statement

In conducting this research, the authors adhered to the “Guide to the Care and Use of Experimental Animals” and “The Ethics of Animal Experimentation” published by the Canadian Council on Animal Care and were approved by the Animal Care Committee at DRDC Suffield Research Centre.

## Author Contributions

YW and TS were responsible for the overall study design and oversaw all experiments. GH and JB were responsible for immunohistochemical experiments. YT and TW carried out electrophysiology experiments. CF performed western blot analysis. TJ carried out blast experiments. TPW and PN were responsible for daily handling, observation, and testing of animals.

## Conflict of Interest Statement

The authors declare that the research was conducted in the absence of any commercial or financial relationships that could be construed as a potential conflict of interest.
